# Comparative Physiological and Transcriptome Analysis of *Crossostephium chinense* Reveals Its Molecular Mechanisms of Salt Tolerance

**DOI:** 10.3390/ijms242316812

**Published:** 2023-11-27

**Authors:** Yuxin Wang, Miao Liu, Ziyu Guo, Yilin Liang, Yufan Lu, Yuxian Xu, Ming Sun

**Affiliations:** State Key Laboratory of Efficient Production of Forest Resources, Beijing Key Laboratory of Ornamental Plants Germplasm Innovation and Molecular Breeding, National Engineering Research Center for Floriculture, Beijing Laboratory of Urban and Rural Ecological Environment, Key Laboratory of Genetics and Breeding in Forest Trees and Ornamental Plants of Ministry of Education, School of Landscape Architecture, Beijing Forestry University, Beijing 100083, China; wangyuxinbjfu@163.com (Y.W.); liumiao@bjfu.edu.cn (M.L.); gzybfu@bjfu.edu.cn (Z.G.); elaineleung@bjfu.edu.cn (Y.L.); lyf1997@bjfu.edu.cn (Y.L.); xuyuxu03@163.com (Y.X.)

**Keywords:** salt stress, *Crossostephium chinense*, ABA signaling transduction, molecular mechanism

## Abstract

*Crossostephium chinense* is a wild species with strong salt tolerance that has great potential to improve the salt tolerance of cultivated chrysanthemums. Conversely, the unique salt-tolerant molecular mechanisms of *Cr. chinense* are still unclear. This study performed a comparative physiological and transcriptome analysis of *Cr. chinense*, *Chrysanthemum lavandulifolium*, and three hybrids to investigate the salt-tolerant molecular mechanisms of *Cr. chinense*. The physiological results showed that *Cr. chinense* maintained higher superoxide dismutase (SOD) activity, alleviating oxidative damage to the membrane. KEGG enrichment analysis showed that plant hormone signaling transduction and the MAPK signaling pathway were mostly enriched in *Cr. chinense* and hybrids under salt stress. Further weighted gene co-expression network analysis (WGCNA) of DEGs suggested that abscisic acid (ABA) signaling transduction may play a significant role in the salt-tolerant mechanisms of *Cr. chinense* and hybrids. The tissue-specific expression patterns of the candidate genes related to ABA signaling transduction and the MAPK signaling pathway indicate that genes related to ABA signaling transduction demonstrated significant expression levels under salt stress. This study offers important insights into exploring the underlying salt-tolerant mechanisms of *Cr. chinense* mediated by ABA signaling transduction and broadens our understanding of the breeding strategies for developing salt-tolerant cultivars utilizing salt-tolerant chrysanthemum germplasms.

## 1. Introduction

The accumulation of salts in soils is a problem that has limited plant growth and productivity [1]. More seriously, salt stress brings out the appearance of wilting and death in plants [2]. For instance, salt stress has both osmotic and ionic effects on plant cells, including secondary effects such as oxidative stress and damage to cellular components [3].

Plants have the ability to defend themselves against varieties of abiotic stresses, activating various responses to maintain their normal growth [4]. Under salt stress, ion imbalance and water deficiency in a plant cell cause osmotic stress, leading to the accumulation of osmolytes and antioxidants [5]. Correspondingly, plants can figure out these different stresses through protein kinases, which are involved in cellular regulation and metabolism. Recent studies suggest that protein kinases such as mitogen-activated protein kinase (MAPK) cascades, calcium-dependent protein kinases (CDPKs/CPKs), and sucrose nonfermenting 1 (SNF1)-related protein kinases (SnRKs) are vital to salt stress-induced oxidative stress response [6,7,8]. However, MAPK activation, which is a secondary response of the change in cellular physiology in plants under stress, is weaker than that induced by pathogen infection under abiotic stress. MAPKs may play an important part in plant abiotic stress response by participating in the biosynthesis or signaling of plant hormones [9]. Moreover, plants have developed phytohormone-mediated stress-tolerant mechanisms [5]. Different mechanisms are exploited through the regulation of stress-responsive gene expression to avoid and tolerate dehydration, which involves ABA and other signaling pathways [10]. Furthermore, MAPKs are reported to be involved in ABA signaling and H_2_O_2_-mediated stomatal closure, indicating a potential link between MAPK cascades with ABA signaling [11].

Chrysanthemum is one of the most important ornamental plants in the world, but the planting areas of cultivated chrysanthemums are limited due to their susceptibility to salt stress. Therefore, improving salt tolerance is an important goal in chrysanthemum breeding. Previous studies have found that some wild species in *Artemisia* and *Chrysanthemum* are more tolerant to salt stress [12,13,14]. Several studies have shown that the salt tolerance of wild chrysanthemums can be inherited by hybrids through distant hybridization [15,16]. According to recent research, genes encoding proteins involved in ABA signaling, ROS scavenging, ion transport (Na^+^, K^+^, and Ca^2+^ transport), and osmotic regulation are all impacted when a chrysanthemum is subjected to salt stress [17,18,19]. Moreover, transcription factor (TF) families in chrysanthemums, including *WRKYs*, *NACs*, *ZIPs*, and *MBFs*, are reported to have important roles in salt stress [20,21,22,23].

*Crossostephium chinense*, a diploid species with strong salt tolerance in the Asteraceae family, is often distributed in the southeast of China [24,25]. We obtained several distant hybrids of *Cr. chinense* and *C. lavandulifolium* in our previous research [26]. In this study, to deeply reveal the unique salt-tolerant mechanisms of *Cr. chinense*, hub genes were selected through physiological, transcriptome, and bioinformatics analysis. Tissue expression analysis revealed that hybrids, like *Cr. chinense* could tolerate high salinity, owing to the function of ABA-related genes under salt stress. Our findings not only provide a better insight into the difference in salt tolerance between *Cr. chinense* and *C. lavandulifolium* but also contribute to potential pathways for breeding better salt-tolerant chrysanthemums.

## 2. Results

### 2.1. The Activities of Antioxidant-Related Enzymes

**Figure 1:**
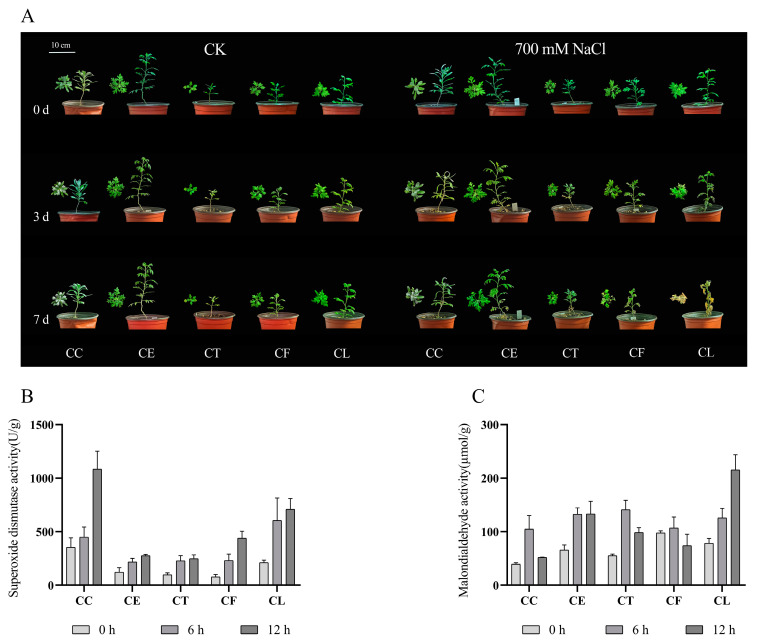
The effect of 700 mM NaCl exposure on the growth and enzymatic antioxidant activities of *Cr. chinense* (CC), *C. lavandulifolium* (CL), and three hybrids (CE, CT, and CF): (**A**) phenotypic differences, (**B**) SOD, (**C**) MDA. Data are means of three replicates and follow normal distribution.

After treatment with 700 mM NaCl for seven days, the growth of *Cr. chinense* (CC) and its hybrids (CE, CT, and CF) was significantly better than *C. lavandulifolium* (CL) (Figure 1A). The results showed that the salt treatment aggravated the wilting of *C. lavandulifolium* due to the accumulation of salinity, whereas *Cr. chinense* and hybrids were only slightly affected. To investigate the various response of five plant materials under salt stress, the activity of SOD and the content of MDA were monitored at three time points. The SOD activity of *Cr. chinense*, *C. lavandulifolium*, and the hybrids increased after 12 h under salt stress (Figure 1B). Furthermore, the SOD activity of CC was substantially higher than that of CL and the three hybrids at 12 h. The MDA content of CC and the three hybrids was obviously less than that of CL, and CC maintained a minimum level (Figure 1C). The results indicate that *Cr. chinense* performed higher antioxidant enzyme activities, preventing oxidative damage to the membrane. In conclusion, the antioxidant enzyme activities of five plant materials began to change after 6 h under 700 mM NaCl treatment, suggesting that the genes related to salt stress were activated.

### 2.2. RNA Sequencing and De Novo Assembly

**Figure 2:**
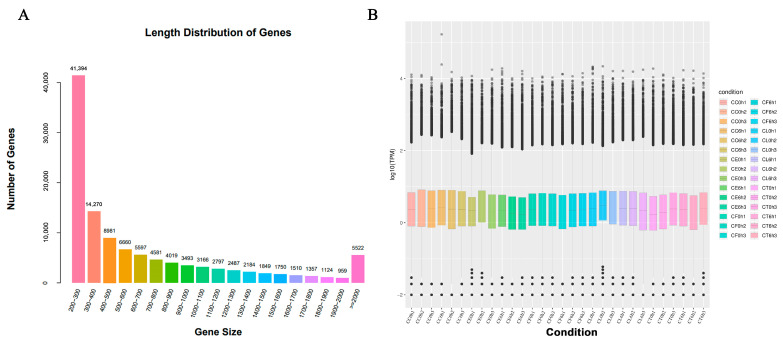
Sequencing data and differentially expressed gene (DEG) results: (**A**) length distribution of genes, (**B**) FPKM box diagram of all tested samples.

To further reveal the molecular mechanisms of *Cr. chinense*, leaf samples treated with 700 mM for 6 h were sequenced for analysis. After filtering the low-quality sequences and the adaptor sequences, a total of 1,200,999,186 clean reads remained, resulting in 168.16 Gb sequence data of 30 libraries. The statistics of the sequencing quality are provided in Table S1 and the de novo assembly statistics are presented in Table S2. The unigene length distribution is shown in Figure 2A, and the three biological replicates of each treatment were similar in the overall gene expression levels (Figure 2B). A total of 113,700 unigenes were annotated after assembly. The details of the unigenes annotated by each database are shown in Table S3.

### 2.3. Comparative Enrichment Analyses of the DEGs under Salt Treatment

**Figure 3:**
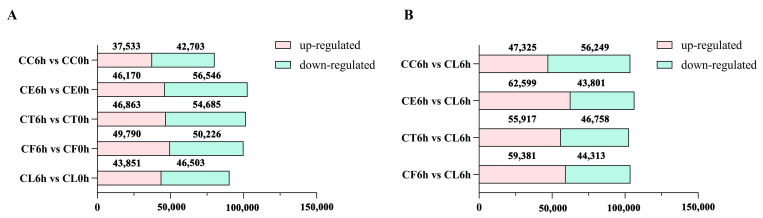
The number of upregulated and downregulated DEGs in all comparison combinations: (**A**) The statistics results of DEGs in comparison of 6h vs 0h. (**B**) The statistics results of DEGs in comparison of 6h vs 6h.

Upon comparing the salt treatment group with the control group, it was noticed that there were more downregulated DEGs in the five samples under salt stress and fewer upregulated genes in *Cr. chinense* than in the hybrids and *C. lavandulifolium* (Figure 3A). In the comparison of the salt treatment group to the salt treatment group of CL, there were more downregulated genes in *Cr. chinense* (Figure 3B). DEGs were further analyzed to compare the different pathways of salt tolerance in the plant materials. To validate the authenticity of the RNA-seq data, the expression levels of 10 randomly selected genes were detected by qRT-PCR. The qRT-PCR results were generally consistent with the DEG analysis of the transcriptome (Figure S1).

**Figure 4:**
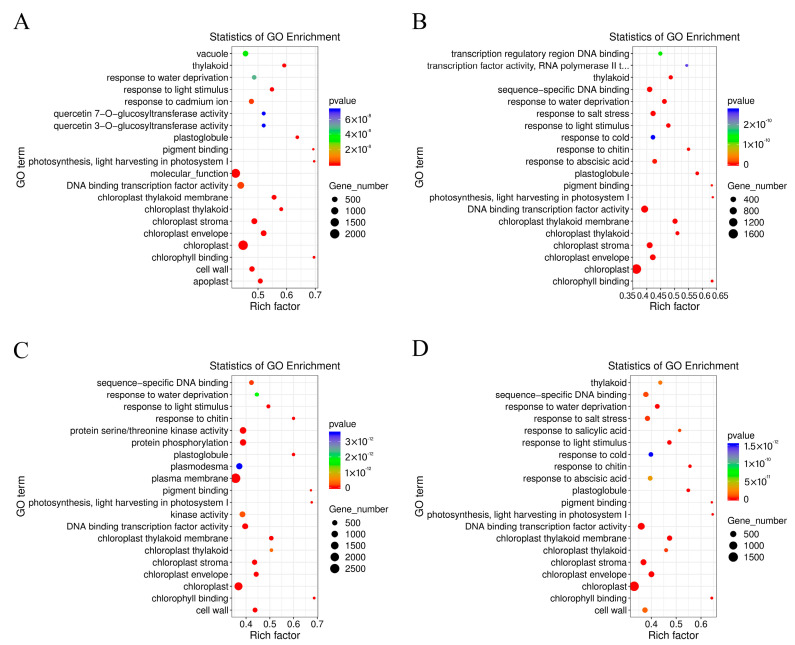
Enrichment analysis of the GO annotation of DEGs in *Cr. chinense*, *C. lavandulifolium*, and hybrids: (**A**) CC6h_vs_CL6h, (**B**) CE6h_vs_CL6h, (**C**) CT6h_vs_CL6h, (**D**) CF6h_vs_CL6h.

**Figure 5:**
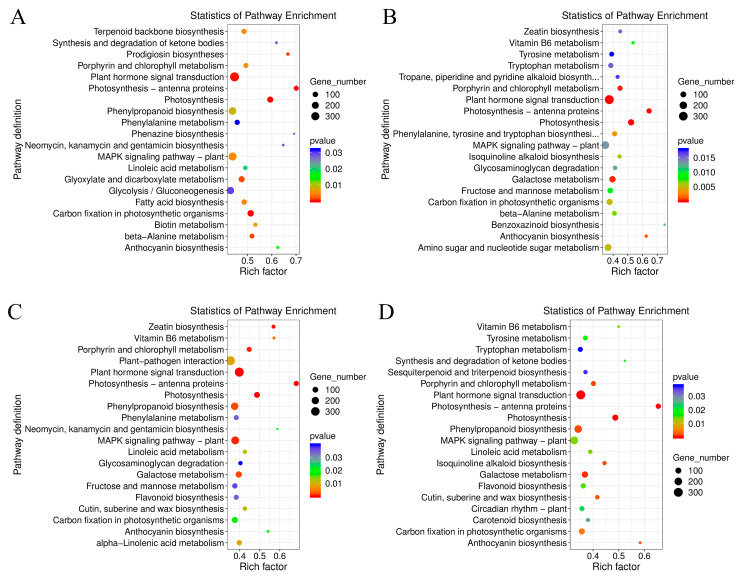
Enrichment analysis of the KEGG pathway of DEGs in *Cr. chinense*, *C. lavandulifolium*, and hybrids: (**A**) CC6h_vs_CL6h, (**B**) CE6h_vs_CL6h, (**C**) CT6h_vs_CL6h, (**D**) CF6h_vs_CL6h.

The GO terms of *C. lavandulifolium* were compared with the salt-tolerant plants under salt stress. The DEGs in four groups were mostly enriched in the “molecular function (GO:0003674)” and “DNA binding transcription factor activity (GO:0003700)” categories. Additionally, the DEGs were especially enriched in the “protein serine/threonine kinase activity (GO:0004674)” term of CT6h_vs_CL6h, the “response to abscisic acid (GO:0009737)” and “response to salt stress (GO:0009651)” terms of CE6h_vs_CL6h, and the “response to water deprivation (GO:0009414)” term of CF6h_vs_CL6h. The results indicate that the response of *Cr. chinense* and hybrids to salt stress involved multiple processes, such as kinase activity and chloroplast activity (Figure 4). Moreover, KEGG-based DEG enrichment analysis showed that plant hormone signal transduction was mostly enriched in four groups under salt stress. Common categories of high enrichment included “photosynthesis” in CC6h_vs_CL6h, “MAPK signaling pathway,” “phenylpropanoid biosynthesis” and “photosynthesis” in CT6h_vs_CL6h, and “galactose metabolism” in CE6h_vs_CL6h (Figure 5). According to the KEGG results, plant hormone signal transduction and the MAPK signaling pathway may be the biological pathways to resistant salt stress in *Cr. chinense* and hybrids.

### 2.4. Analysis of Gene Co-Expression Network

**Figure 6:**
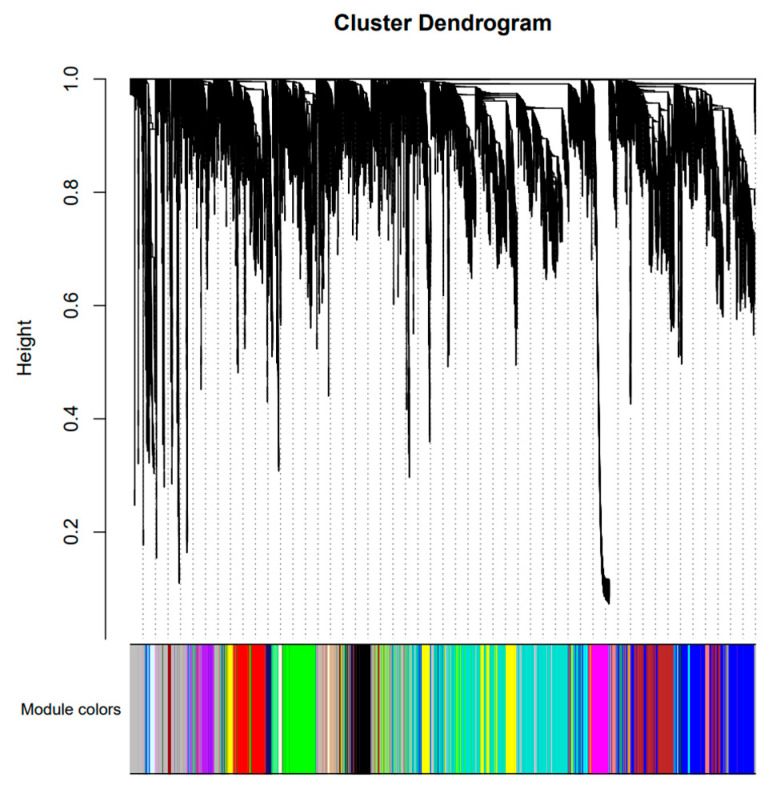
Gene cluster dendrograms and module division.

To further understand the gene expression of CC, CL, and the three hybrids under salt stress, the hub genes related to salt tolerance were screened out through weighted gene co-expression network analysis (WGCNA). After filtering raw data in materials and methods, 34,760 genes were retained for WGCNA analysis. The co-expression network was constructed based on the gene expression of 30 samples. In this study, we selected the weight value β = 22 to construct scale-free networks, describing different modules with different colors. A total of 35 modules were identified after identifying the result of the system clustering tree via dynamic hybrid algorithm based on dissTOM (Figure 6). The blue module (5041) had the maximum count, and the dark magenta module had the minimum count (40) (Table S4).

**Figure 7:**
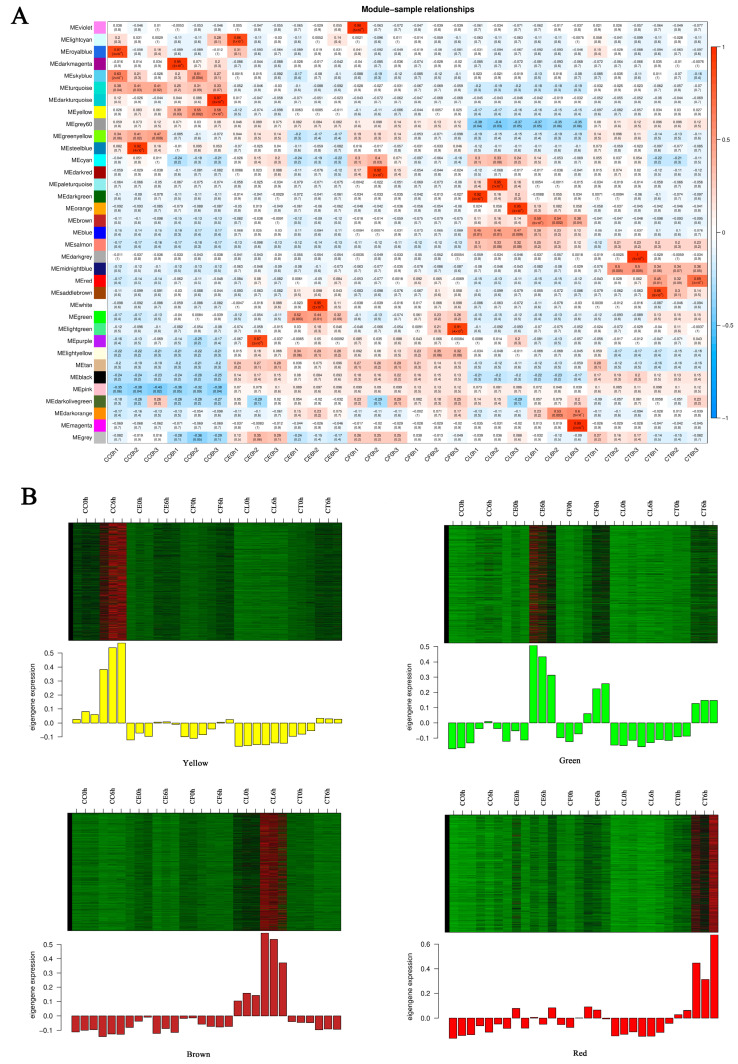
Sample expression heatmap of co-expression modules. (**A**) The color in the heatmap represents the module’s characteristic value, with red representing high expression and blue representing low expression. (**B**) The above figure is the expression heatmap of genes in the module in different samples, and the figure below shows the eigenvalues of modules in different samples, with red indicating upregulation and green indicating downregulation.

To screen out modules that were significantly related to specific samples, a heatmap of the sample expression mode was conducted to select the corresponding modules. As shown in the picture (Figure 7A), the yellow module had relatively high correlation with CC6h, indicating that the genes in the yellow module may have been related to salt stress response. Similarly, the green module was highly correlated with CE6h and CF6h, the red module was highly correlated with CT6h, and the brown module was highly correlated with CL6h. Therefore, this study identified four co-expression modules as key modules for further research. Analysis of the expression patterns of the genes showed that the genes in each module were differentially expressed in different samples (Figure 7B). Notably, the genes in the green module were upregulated in CE and CF, and the genes in the yellow module were especially upregulated in CC.

KEGG pathway enrichment analysis showed that the highly enriched pathways in the environmental information processing of four modules included plant hormone signal transduction (map04075) and the MAPK signaling pathway (map04016). The GO enrichment analysis showed that the highly enriched biological processes in the green module included response to water deprivation (GO:0009414) and response to abscisic acid (GO:0009737). In the yellow and red modules, the biological processes were differentially enriched in protein phosphorylation (GO:0006468) and the defense response (GO:0006952) (Figure S2). The results indicate that the MAPK signaling pathway and plant hormone signaling transduction, particularly abscisic acid signaling transduction, played an important role in the response of *Cr. chinense* and its hybrids to salt stress.

**Figure 8:**
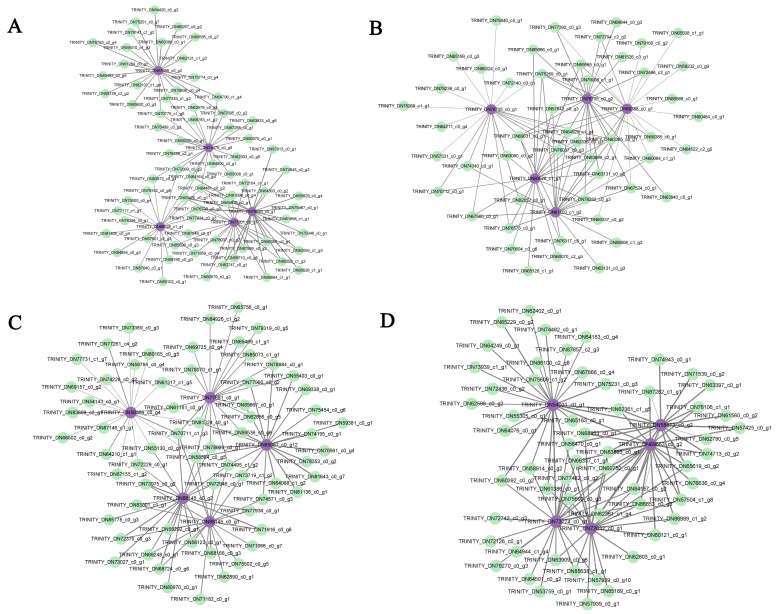
Regulatory network diagram of hub genes and associated genes in key modules. Purple represents the hub genes; green represents the top 20 genes associated with the hub genes. (**A**) The co-expression network genes in the yellow module. (**B**) The co-expression network genes in the green module. (**C**) The co-expression network genes in the brown module. (D) The co-expression network genes in the red module.

Plant hormone signal transduction and the MAPK signaling pathway were merged in each module, and the genes enriched in each module were sorted according to the order of gene connectivity from high to low. The top five genes with gene connectivity were regarded as hub genes (Table S5), and the top 20 genes with weight values from high to low related to the hub genes were considered the associated genes (Table S6). In the yellow module, 71 associated genes were screened, including *TRINITY_DN84481_c0_g2*, which encoded TATA-box-binding protein-associated factor (TAF), and *TRINITY_DN65102_c0_g1*, which encoded trehalose-6-phosphate synthase (TPS) and genes encoding flavonoid biosynthesis, DNA binding, and transporter activity. Most of associated genes were screened as unknown genes. In the green module, 49 associated genes were screened, including *TRINITY_DN83699_c2_g1*, encoding 9-cis-epoxycarotenoid dioxygenase3 (NCED3); *TRINITY_DN59565_c0_g1*, encoding calcineurin B-like calcium sensor interacting protein kinase (CIPK); and *TRINITY_DN70712_c0_g1*, encoding MAPK kinases and genes encoding protein serine/threonine phosphatase activity and the plasma membrane. In the brown module, 64 associated genes were screened, including genes encoding protein binding and chlorophyll binding. In the red module, 56 associated genes were screened, including genes encoding DNA binding and calcium ion binding (Table S7). The weighted gene co-expression network was drawn using Cytoscape_3.9.1 (Figure 8).

### 2.5. Tissue-Specific Expression Pattern Analysis of Hub Genes

**Figure 9:**
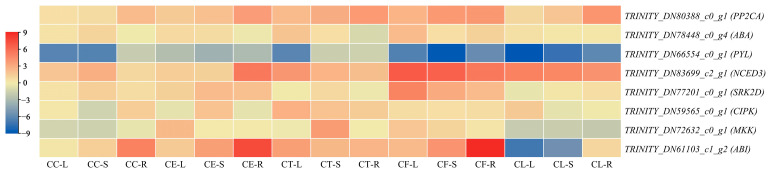
The relative expression heatmap of hub genes under salt treatment, as determined by qRT-PCR, in the leaves (L), shoots (S), and roots (R) of *Cr. chinense* (CC), *C. lavandulifolium* (CL), and three hybrids (CE, CT, and CF). The red color represents high gene expression, and the blue color represents low gene expression.

To further explore the tissue-specific expression patterns of the hub genes under salt stress, qRT-PCR was performed on the leaves, shoots, and roots of *Cr. chinense*, *C. lavandulifolium*, and hybrids (Figure 9). Under salt stress, the gene *TRINITY_DN66554_c0_g1* exhibited a significant downregulation trend, indicating the salt-tolerant mechanisms of activating ABA signaling transduction in *Cr. chinense* and hybrids. Remarkably, the genes related to the ABA signaling pathway yielded high expression levels in the hybrids, indicating their involvement in resisting salt stress. The results of qRT-PCR analysis provided further evidence for revealing the salt-tolerant molecular mechanisms of *Cr. chinense* and hybrids.

## 3. Discussion

Soil salinization is one of the most severe environmental stresses. In order to cope with saline–alkaline land, salt-tolerant genetic resources need to be developed using both traditional and molecular breeding techniques [10]. *Cr. chinense* is a salt-tolerant cultivar, but little is known about its salt-tolerant mechanisms. The intergeneric hybridization between *Cr. chinense* and *C. lavandulifolium* improves the salt tolerance of hybrids. Osmotic stress and ionic stress are two major stresses that affect the growth of plants under high salinity [27]. Recent studies have reported on the critical role of Raf-like kinases (RAFs) in early osmotic stress, mediating osmoregulation in *Arabidopsis thaliana* [28]. Thus, understanding the salt-tolerant strategies of *Cr. chinense* in early osmotic stress can help us explore the salt-tolerant mechanism of *Cr. chinense*. The physiological results showed that *Cr. chinense* maintained higher antioxidant enzyme activity after treatment with 700 mM NaCl, preventing oxidative damage to the membrane. Under high salinity, maintaining high levels of antioxidant enzyme activity may be one of the key elements for *Cr. chinense* and its hybrids to keep growing. In the present study, we performed de novo transcriptome sequencing of *Cr. chinense*, *C. lavandulifolium*, and hybrids to better analyze the underlying molecular mechanisms of strong salt tolerance in *Cr. chinense*. To date, although a few transcriptome studies of salt stress in chrysanthemums have been published [29,30], there is a lack of transcriptome analysis exploring the differences between salt-tolerant cultivars and intergeneric hybrids. The results of GO and KEGG analysis of the whole transcriptome showed that plant hormone signaling transduction and the MAPK signaling pathway featured in the response of *Cr. chinense* and its hybrids to salt stress. The KEGG and GO pathway enrichment in WGCNA analysis further revealed that ABA signaling transduction might play a crucial role in the process of salt tolerance. Thus, we speculate that ABA signaling transduction plays an important role in the defense of *Cr. chinense* against salt stress. The high expression of ABA-related genes in the hybrids demonstrates the salt-tolerant inheritance of the hybrids from *Cr. chinense* and further reveals the significant roles of ABA signaling transduction in the salt tolerance of *Cr. chinense*.

Plant hormones are significant signaling molecules that regulate growth, development, and defense in plants [31]. The five hub genes in the green module, which was highly correlated with the CE and CF samples in terms of salt stress, were all related to the hormone abscisic acid. ABA plays a crucial role in the closure of stomata by regulating guard cell ion fluxes towards varieties of stresses, including salt, drought, and water [32,33,34]. The ABA receptor-coupled core signaling pathway consists of three components containing the PYRABACTIN RESISTANCE (PYR)/PYR-LIKE (PYL)/REGULATORY COMPONENTS OF ABA RECEPTORS (RCAR) family proteins, negative regulator clade A type 2 C protein phosphatases (PP2Cs), and positive regulator SNF1-related protein kinases 2 (SnRK2s) [35]. After ABA binding to PYR/PYL/RCARs, the ABA receptor complex inhibits the activity of PP2C phosphatases, releasing SnRK2s from PP2C-mediated inhibition [36]. SnRK2s are activated through autophosphorylation or other protein kinases, participating in a variety of physiological responses through phosphorylating target substrates, including ion channels, transcription factors, and transporters [37]. PP2CA and ABI1 are two branches of protein phosphatase type 2C (PP2C) from group A [38]. It has been established that ABI1 is a negative regulator of SnRK2.4 and that PP2CA interacts with and inhibits SnRK2.4, which belongs to the SnRK2s activated by ABA [39]. However, the expression levels of PP2Cs are actually upregulated by abiotic stress and ABA, which is possibly induced by an ABA desensitization mechanism to adjust ABA signaling [40]. 

To prevent plants from losing water, ABA can mediate the closure of stomata pores by activating Ca^2+^ entry into the cytoplasm [41,42]. The evidence shows that Ca^2+^ signals can be decoded by several Ca^2+^ sensors, including calcium-dependent protein kinase CPK6, which can activate the downstream targets involved in stomatal closure [43]. In our study, we found that the gene *TRINITY_DN59565_c0_g1* encoding CIPK2 had a correlation with the five hub genes in the green module, which may have been the potential conduction factors in ABA signaling pathway. In addition, the hub gene *TRINITY_DN80528_c1_g1* encoding ABA-response element (ABRE)-binding factors (ABFs) in the green module may have been related to the hub gene *TRINITY_DN77201_c0_g1* in the yellow module. Recent research has shown that PtrSnRK2.4 can interact with PtrABF2 by forming a heterodimer and that PtrABF2 can be phosphorylated by PtrSnRK2.4 at Ser93 to modulate the drought tolerance of plants [44]. Moreover, overexpressing *DcABF3* in Arabidopsis increases the stomatal density and affects the water deficit tolerance, indicating that ABFs can mediate the stomatal development [45]. 

Moreover, the gene *TRINITY_DN83699_c2_g1* encoding 9-cis-epoxycarotenoid dioxygenase 3 (NCED3) as a candidate gene was found to have a strong correlation with the five hub genes in the green module. The plant endogenous abscisic acid levels increase dramatically, which is important to stomatal regulation and gene expression during periods of abiotic stress [46,47]. ABA is metabolized from xanthoxin, which is cleaved from carotenoids 9-cisneoxanthin and 9-cis-violoxanthin in the chloroplast [47]. This carotenoid cleavage reaction is catalyzed by 9-cisepoxycartenoid dioxygenases (NCEDs) [46]. In addition, both ABA-dependent and ABA-independent pathways govern the induction of NCED3, showing that NCED3 is NaCl dependent [48]. In *Arabidopsis*, *AtNCED3* was proven to be the major stress-induced NCED in leaves, and another four *AtNCEDs* (2, 5, 6, and 9) were found to differ in the binding activity of the thylakoid membrane [49]. This evidence indicates that ABA plays a significant role through a variety of ABA receptors and key enzyme genes. PP2Cs played a negative role in the process of plant endogenous ABA levels. Consistent with the previous conclusions, this study found that salt stress activated the expression of key elements in ABA signaling transduction, of which *TRINITY_DN61103_c1_g2*, *TRINITY_DN80388_c0_g1*, *TRINITY_DN80528_c1_g1*, and *TRINITY_DN83699_c2_g1* remain to be confirmed.

There has been a whole train of regulatory processes coordinating plant responses to abiotic stresses in plants, including chromatin modifications, transcriptional regulation, alternative splicing, protein phosphorylation, and ubiquitnation/sumoylation [50]. Among them, protein phosphorylation is an important mode of signal transduction to plants, where protein kinases play a crucial role in transferring the phosphoryl group [51,52]. In this study, the MAPK signaling pathway was significantly enriched in the red module, which was highly related to the CT sample in terms of salt stress. Mitogen-activated protein kinase modulates plant tolerance to salt stress, and the canonical MAPK is composed of three types of kinases: MAPK kinase kinases (MAPKKKs/MAP3Ks/MEKKs), MAPK kinases (MKKs/MAP2Ks/MEKs), and MAP kinases (MAPKs/MPKs) in a sequential phosphorelay starting from a MAPKKK [53,54]. Evidence shows that MAPK cascades like MAP3K17/18, MKK3, and MPK1/2/7/14 are activated by the PYR/PYL/RCAR-SnRK2-PP2C ABA core signaling module through protein synthesis of the MAP3Ks [55]. Furthermore, MPK1 and MPK2 can be activated by ABA in a SRK2D/E/I-dependent manner, connecting the ABA and MAPK modules [56]. In *Arabidopsis*, MPK6 can interact with and phosphorylate the C-terminal fragment of SOS1, which can extrude Na^+^ into the soil solution and load Na^+^ into the xylem [57]. Accordingly, we found that the gene *TRINITY_DN77201_c0_g1* in the yellow module, which was highly associated with CC, and the gene *TRINITY_DN75923_c1_g1* in the brown module, which was associated with CL, were related to the SnRK2 gene family. In conclusion, it is likely that the MAPK signaling pathways, which may function in ABA signaling transduction through potential genes, regulate the salt tolerance of *Cr. chinense*. These pathways will need to be further investigated in future studies.

**Figure 10:**
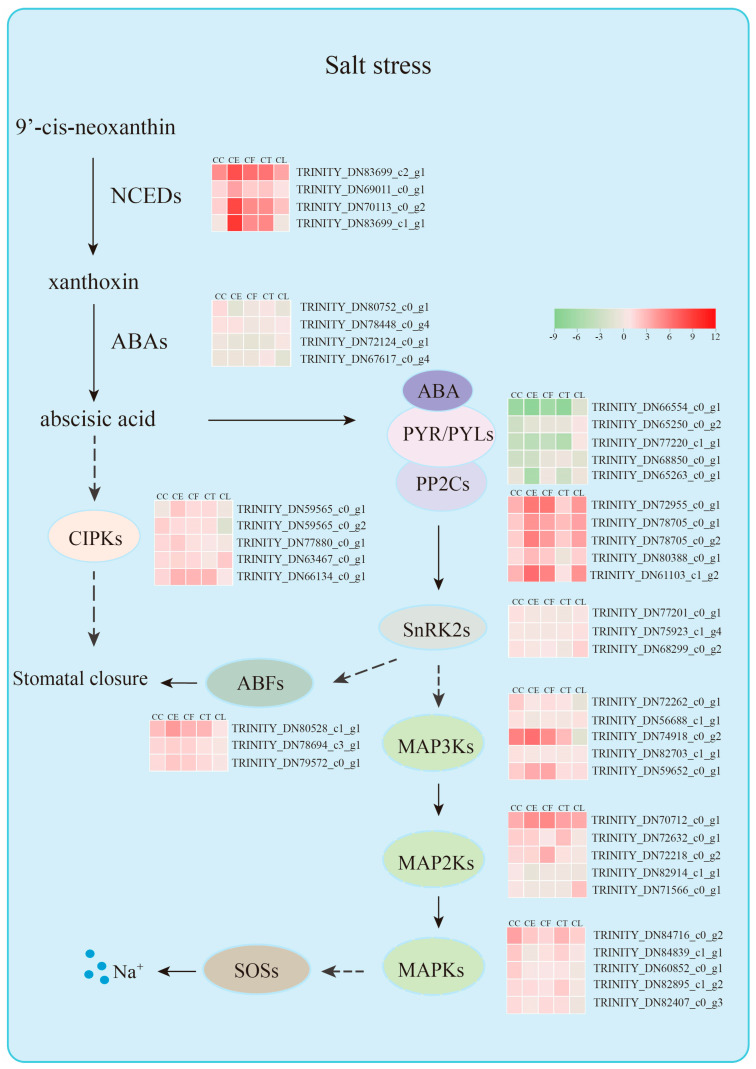
Molecular mechanism diagram of salt stress resistance of *Cr. chinense*.

Tissue-specific expression patterns of candidate genes could provide better knowledge of the gene functions in different plant materials. The genes *TRINITY_DN66554_c0_g1* and *TRINITY_DN83699_c2_g1* showed significant expression levels the in leaves, shoots, and roots, indicating that the carotenoid pathway was activated to synthesize the endogenous ABA that positively regulated salt stress in *Cr. chinense* and hybrids. Two highly expressed genes in the hybrids, *TRINITY_DN80388_c0_g1* and *TRINITY_DN61103_c1_g2*, revealed salt-tolerant mechanisms similar to those of *Cr. chinense*. Collectively, these findings indicate that, under salt stress, the hybrids employed the same salt-tolerant mechanisms as *Cr. chinense*, with the ABA signaling pathway playing a significant role. Based on the hub genes and other potential associated genes screened in each module, a hypothetical model is proposed to explain the mechanism of *Cr. chinense* under salt stress and analyze the differential expression of the gene family in related pathways (Figure 10). However, further studies are required to validate the definite roles of the genes and the potential connections between the signaling pathways.

## 4. Materials and Methods

### 4.1. Plant Materials and Culture Conditions

*Cr. chinense* (CC), *C. lavandulifolium* (CL), and the three hybrids (CE, CT, CF) were conserved in the breeding greenhouse of the National Flower Engineering Center, Beijing, China. Rooted seedlings at the 6–7 leaf stage were transplanted into a soil mixture of peat, vermiculite, and pearlite (3:1:1, *v*/*v*/*v*). The plants were cultured at 22 ± 2 °C and 65 ± 5% relative humidity with a photoperiod of 16:8 (light:dark). Uniform plant seedlings with 8–10 leaves were selected for the subsequent experiment.

### 4.2. Salt Treatments and Stress Tolerance Observation

In our previous investigation, *Cr. chinense* was shown to be highly salt tolerant, with a salt tolerance of up to 700 mmol/L, and its hybrids inherited this high tolerance. To observe the salt-tolerant differences in *Cr. chinense*, *C. lavandulifolium*, and the hybrids, the plant materials were irrigated with 700 mM NaCl. All of the plant materials were photographed on day 3 and day 7.

To ensure the best time point for transcriptome sampling, the seedlings were treated with 700 mM NaCl at 8 am. Fresh leaves (about 0.1 g) were harvested after 0, 6, and 12 h of treatment to determine the SOD and the MDA activity. The activities of SOD (U/g FW) and MDA (µmol/g FW) were measured using analysis kits (Nanjing Jiancheng Bioengineering Institute, Nanjing, China, www.njjcbio.com) (accessed on 20 June 2021).

### 4.3. cDNA Library Construction and Illumina Sequencing

For transcriptome analysis, the plant materials were exposed to 700 mM NaCl for 6 h. All leaf samples of each set with three biological replicates were immediately immersed in liquid nitrogen and stored at −80 °C for RNA extraction. Total RNA was extracted from the plant leaves using TRIzol reagent (Invitrogen, Waltham, CA, USA) following the manufacturer’s procedure. To analyze the quantity and purity of the total RNA, the Bioanalyzer 2100 and RNA 1000 Nano LabChip Kit (Agilent, Santa Clara, CA, USA) with RIN number > 7.0 were used. Poly (A) RNA was purified from total RNA (5 µg) using poly-T oligo-attached magnetic beads and two rounds of purification. Following purification, the mRNA was fragmented into small pieces using divalent cations under elevated temperature. The cleaved RNA fragments were reverse-transcribed to create the final cDNA library in accordance with the protocol for the mRNASeq sample preparation kit (Illumina, San Diego, CA, USA). The average insert size for the paired-end libraries was 300 bp (±50 bp). The paired-end sequencing was performed on an Illumina Novaseq^TM^ 6000 (LC Sciences, Houston, TX, USA) following the vendor’s recommended protocol.

### 4.4. De Novo Assembly, Unigene Annotation, and Functional Classification

Clean reads were obtained after using the Cutadapt and perl scripts in house to remove the reads that contained adaptor contamination, low-quality bases, and undetermined bases [58]. Then, the sequence quality was verified using FastQC (http://www.bioinformatics.babraham.ac.uk/projects/fastqc/) (accessed on 16 January 2022), based on the Q20, Q30, and GC content of the clean data. All downstream analyses were based on clean data of high quality. De novo assembly of the transcriptome was performed with Trinity 2.4.0 [59]. For shared sequence content, trinity group transcripts were clustered into unigenes. All assembled unigenes were aligned with the non-redundant (Nr) protein database (http://www.ncbi.nlm.nih.gov/) (accessed on 16 January 2022) and the Gene Ontology (GO) (http://www.geneontology.org) (accessed on 16 January 2022), SwissProt (http://www.expasy.ch/sprot/) (accessed on 16 January 2022), Kyoto Encyclopedia of Genes and Genomes (KEGG) (http://www.genome.jp/kegg/) (accessed on 16 January 2022), and eggNOG (http://eggnogdb.embl.de/) (accessed on 16 January 2022) databases using DIAMOND with an E-value threshold < 0.00001 [60].

### 4.5. Differentially Expressed Unigene Analysis

To determine the expression level of the unigenes, salmon was used by calculating the TPM [61,62]. The differentially expressed unigenes were selected with a log2 (fold change) > 1 or a log2 (fold change) < −1 and with statistical significance (*p*-value < 0.05) with the R package edgeR [63]. GO and KEGG enrichment analyses were performed on the differentially expressed unigenes using perl scripts in house. Ten randomly selected DEGs were used for qRT-PCR analysis to validate the authenticity of the RNA-seq data.

### 4.6. Weighted Gene Co-Expression Network Analysis

Co-expression-network analysis was constructed using the weighted gene correlation network analysis (WGCNA) package in R under the guidelines of the published tutorials. The high-quality genes were screened according to the standard of FPKM ≥ 10. Hierarchical clustering of the samples was conducted based on Euclidean distance computed on gene expression data and integrated with the clinical information of patients. Outlier samples were removed. The parameters were selected by default software parameters (soft threshold = 22 (estimate value); min module size = 30; merge cut height = 0.25). The core DEGs were further divided into 36 modules. For each module, the eigengene (the first component expression of the genes in the module) was determined, and the correlations of the eigengenes were then subsequently calculated. Genes with high connectivity in the respective modules were considered hub genes. Network visualization for each module was performed using Cytoscape software version 3.9.1, with the weight parameter cut-off, obtained from the WGCNA, set at 0.3.

### 4.7. Tissue-Specific Expression Patterns of the Candidate Genes

To analyze the gene expression of different tissues in plants, 8 candidate genes with high expression in key modules were selected for qRT-PCR analysis. The experimental plants were exposed to 700 mM NaCl for 6 h. The collecting methods of leaf, shoot, and root samples were consistent to with a value of 2.3. The specific primers for these 8 genes and the actin gene (an internal control) were designed by IDT (https://sg.idtdna.com/PrimerQuest/Home/Index) (accessed on 16 January 2022) (Table S8). One µg of total RNA was used to synthesize the cDNA according to the manufacture’s instruction with the PrimeScript^TM^ RT reagent kit and gDNA Eraser Perfect Real Time (Takara, Dalian, China). The qRT-PCR reaction (10 µL) was formulated using the TB Green Premix Ex Taq II (Takara, Dalian, China). All qRT-PCRs were carried out on a Piko^®^ Thermal Cycler 96-well system (Thermo Scientific, Waltham, MA, USA). The experimental methods of qRT-PCR were adapted to a previous study [64]. The average threshold cycle (CT) from three biological replicates was employed to calculate the gene expression fold change via the 2^−△△Ct^ method. The results of the tissue-specific expression were visualized by heatmap using TBtools [65].

## 5. Conclusions

*Cr. chinense* is a salt-tolerant wild species, and its hybrids inherit this strong tolerance. The comparative transcriptome analyses indicated that plant hormone signaling transduction and the MAPK signaling pathway were the key biological pathways. Further WGCNA analysis shed light on the role of ABA signaling transduction and its underlying interaction with the MAPK signaling pathway in the modulation of the salt tolerance of *Cr. chinense*. The tissue-specific expression patterns of candidate genes showed that the ABA-related genes were significantly expressed under salt stress. These candidate genes and pathways uncovered the intricate salt-tolerant mechanisms with which ABA signaling transduction may regulate the salt tolerance of *Cr. chinense* through interaction with the MAPK signaling pathway, with significant implications for exploring and developing salt-tolerant chrysanthemum germplasms.

## Data Availability

The data presented in this study are available in Supplementary Materials.

## Supplementary Materials

The following supporting information can be downloaded at: https://www.mdpi.com/article/10.3390/ijms242316812/s1.
