# Supplementary figures and images for: Comparative Physiological and Transcriptome Analysis of Crossostephium chinense Reveals Its Molecular Mechanisms of Salt Tolerance
Source: Int J Mol Sci. 2023 Nov 27;24(23):16812. doi: 10.3390/ijms242316812 (PMC10706559; doi:10.3390/ijms242316812)

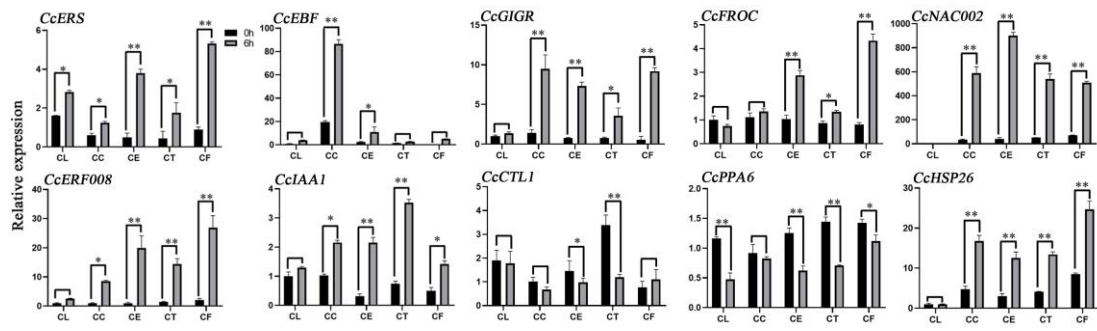

Figure S1.

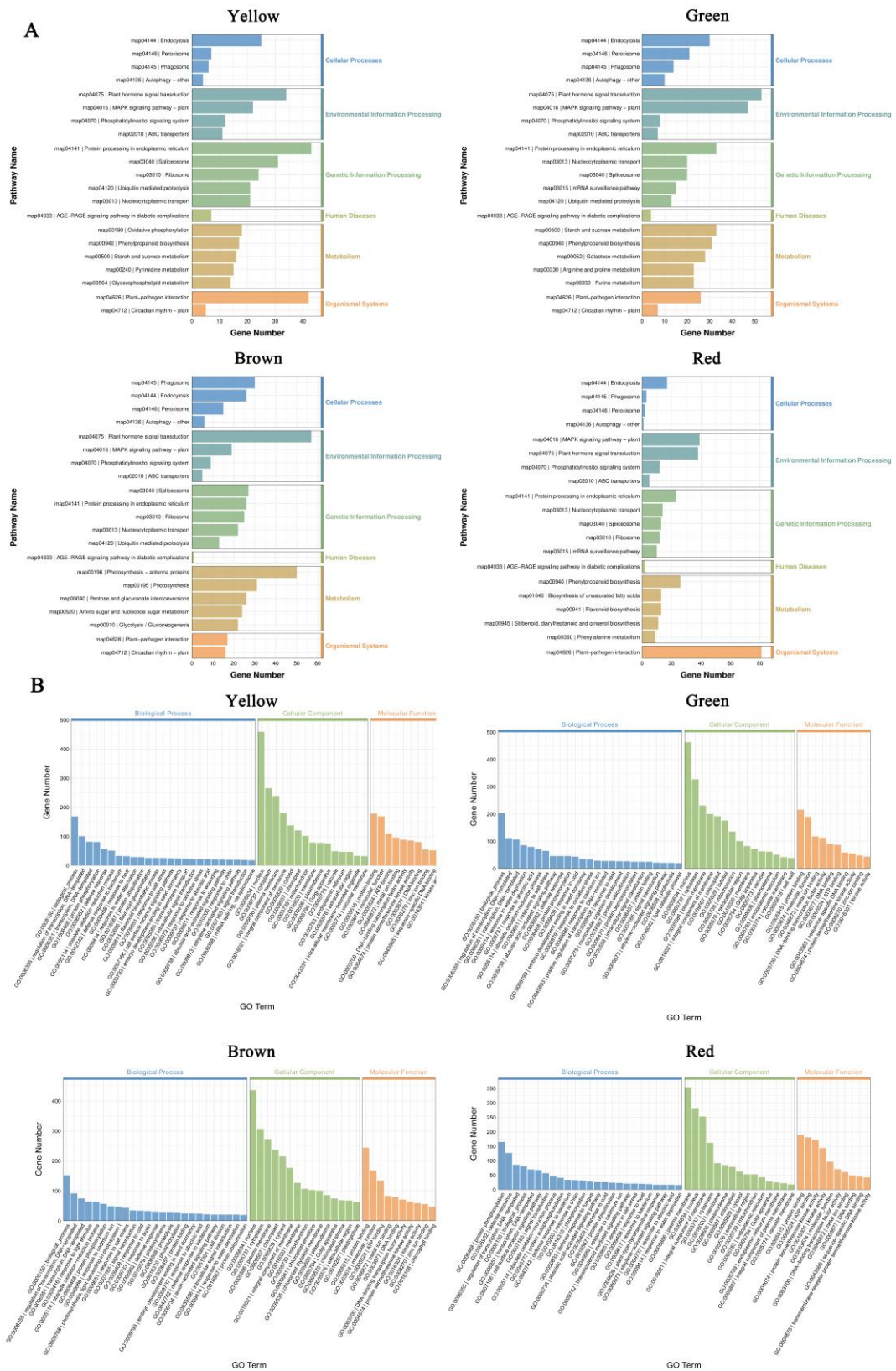

Figure S2.

Supplement: Supplementary file 1 [file ijms-24-16812-s001.zip › ijms-2665907- Figure S1 and S2..pdf]
